# Improving usability benchmarking for the eHealth domain: *The development of the eHealth UsaBility Benchmarking instrument (HUBBI)*

**DOI:** 10.1371/journal.pone.0262036

**Published:** 2022-02-17

**Authors:** Marijke Broekhuis, Lex van Velsen

**Affiliations:** 1 Roessingh Research and Development, eHealth Group, Enschede, The Netherlands; 2 Biomedical Signals and Systems, Faculty of Electrical Engineering, Mathematics and Computer Science (EEMCS), University of Twente, Enschede, The Netherlands; Cape Peninsula University of Technology, SOUTH AFRICA

## Abstract

**Background:**

Currently, most usability benchmarking tools used within the eHealth domain are based on re-classifications of old usability frameworks or generic usability surveys. This makes them outdated and not well suited for the eHealth domain. Recently, a new ontology of usability factors was developed for the eHealth domain. It consists of eight categories: Basic System Performance (BSP), Task-Technology Fit (TTF), Accessibility (ACC), Interface Design (ID), Navigation & Structure (NS), Information & Terminology (IT), Guidance & Support (GS) and Satisfaction (SAT).

**Objective:**

The goal of this study is to develop a new usability benchmarking tool for eHealth, the eHealth UsaBility Benchmarking Instrument (HUBBI), that is based on a new ontology of usability factors for eHealth.

**Methods:**

First, a large item pool was generated containing 66 items. Then, an online usability test was conducted, using the case study of a Dutch website for general health advice. Participants had to perform three tasks on the website, after which they completed the HUBBI. Using Partial Least Squares Structural Equation Modelling (PLS-SEM), we identified the items that assess each factor best and that, together, make up the HUBBI.

**Results:**

A total of 148 persons participated. Our selection of items resulted in a shortened version of the HUBBI, containing 18 items. The category Accessibility is not included in the final version, due to the wide range of eHealth services and their heterogeneous populations. This creates a constantly different role of Accessibility, which is a problem for a uniform benchmarking tool.,

**Conclusions:**

The HUBBI is a new and comprehensive usability benchmarking tool for the eHealth domain. It assesses usability on seven domains (BSP, TTF, ID, NS, IT, GS, SAT) in which a score per domain is generated. This can help eHealth developers to quickly determine which areas of the eHealth system’s usability need to be optimized.

## Introduction

**Fig 1:**
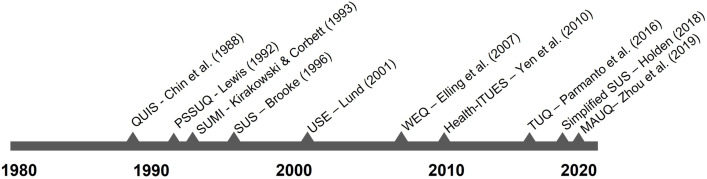
Timeline of the development of usability questionnaires.

Usability testing is an important part of the design process of an eHealth service. It allows developers to understand how they can improve the interface and interaction design of their technology. Most often, such a test is accompanied by assessing, or benchmarking, the overall usability of the eHealth service. Of all usability benchmarking tools, The System Usability Scale (SUS) is the most popular in the eHealth domain [1]. However, the generic nature of the SUS is a large drawback. It does not consider the domain-specific factors that shape the usability of an eHealth service (e.g., does a patient understand the technical jargon that is used in the service?). This tendency to use general benchmarking instruments for assessing eHealth usability has been common practice for years, and has led to use of generic instruments, such as the SUS [2], Questionnaire for Usability of Interface Satisfaction (QUIS) [3], Post-Study System Usability Questionnaire (PSSUQ) [4], SUMI [5] and the Usefulness, Satisfaction and Ease of use (USE) questionnaire [6]. These benchmarking tools were developed in the early days of the field of human-computer interaction (see Fig 1). During this period, it was thought that the same rules for good usability apply for every product, system or service. As a consequence, general usability benchmarking tools were developed that were technology-agnostic. This perspective on usability remained quite persistent for decades.

Only recently, a growing awareness arose, in which it is acknowledged that the makeup of the concept of usability depends on the type of technology [7–9]. This is especially the case for eHealth services [10]. There are many factors that are specifically related to the health domain, in which eHealth services are embedded, that could (negatively) affect the usability of a system as perceived by the users. Examples of such factors are eHealth services that use complicated medical jargon or that they should take into account that they are sometimes used in times of stress. New usability benchmarking questionnaires have been developed, specifically for the eHealth domain, like the TeleHealth Usability Questionnaire (TUQ) [11], the Health-ITUES [12], and the MAUQ [13]; or generic benchmarking questionnaires were adapted to the eHealth context, like the Simplified SUS scale [14], that is developed for measuring usability of services for cognitively impaired older adults. Fig 1 shows a timeline of the development of usability questionnaires from the eighties up till now.

While the emergence of eHealth-specific instruments is a very positive development, the community seems to have skipped an important step: creating a comprehensive understanding of the usability concept for the eHealth context. A recent study proposed a new ontology of usability factors, specifically for the eHealth domain [15], based on a content analysis of 400 usability problems found in eight contemporary eHealth services. These eHealth services cover a wide range of different systems like a gamified exercise module, home monitoring tool, a robotic screening tool, mobile apps and an online coaching platform. The ontology lists a total of thirteen general usability factors and seven eHealth-specific usability factors, clustered into eight main categories: System Basic Performance, Task-Technology Fit, Accessibility, Interface Design, Navigation & Structure, Content & Terminology, and Guidance & Support and Satisfaction. Table 1 shows the complete overview of usability factors per category.

**Table 1:** Usability ontology for eHealth (from Broekhuis et al. [15]).

Category	Usability factor	Type of usability factor
**Basic system performance**	Technical performance	General
	General system interaction	General
**Task-Technology Fit**	Fit between system and context of use	General
	Fit between system and user	General
	Fit between system and health goals	eHealth-specific
**Accessibility**	Accommodation to perceptual limitations	eHealth-specific
	Accommodation to physical limitations	eHealth-specific
	Accommodation to cognitive limitations	eHealth-specific
**Interface Design**	Design clarity	General
	Symbols, icons and buttons	General
	Interface organization	General
	Readability of texts	General
**Navigation & Structure**	Navigation	General
	Structure	General
**Information & Terminology**	System information	General
	Health-related information	eHealth-specific
**Guidance & Support**	Error management	General
	Procedural system information	General
	Procedural health-related information	eHealth
**Satisfaction**	Satisfaction with system	General
	Satisfaction with system’s ability to support health goals	eHealth-specific

Existing usability benchmarking instruments are insufficient to assess the usability of an eHealth service because they are incomplete in the usability factors that the instruments assess. They are too generic or too focused on eHealth-specific factors. The ontology takes both into account, identifying both general and eHealth-specific factors that need to be considered when evaluating the usability of eHealth services. The study by Broekhuis et al. [15] further shows how 30% of the usability problems in eHealth services are related to eHealth-specific factors. This means that current, generic usability benchmarking instruments, such as the SUS [2] and the PSSUQ [4] measure only a subset (70% at best) of the general usability factors and ignore eHealth-specific usability factors. In contrast, eHealth-specific instruments, like the MAUQ [13] and the Health-ITUES [12], include only a few generic usability factors. These questionnaires also have another more fundamental problem. They are mainly built or adapted from older benchmarking instruments, like the SUS or the PSSUQ, which are, again, generic instruments. Furthermore, there have been no studies conduct that analyse how these instruments asses the usability of eHealth and how predictive their outcomes are for the number of (crucial) usability problems [8].

The aim of this study is to develop a new usability benchmarking instrument, specifically for the eHealth domain. This instrument is named: “the eHealth UsaBility Benchmarking Instrument” (HUBBI), and is based on the ontology developed by Broekhuis et al. (2020). With the HUBBI, we strive to develop an instrument that is easy and quick to administer and provides insights in how various aspects of system usability are rated by the patients that will use the system. This can help researchers and practitioners in the field of usability, human-computer interaction and system development to quickly determine which elements of the eHealth system needs to be improved before implementation.

## Materials and methods

### Research context

In the Netherlands, the Dutch Federation of General Practitioners has created a website (thuisarts.nl) that aims to minimize the number of unnecessary visits to the General Practitioner by offering self-help advice for minor ailments. Additionally, the website instructs patients when to contact their General Practitioner’s office.

### Benchmark development

We developed a benchmarking instrument based on the theoretical ontology of eHealth usability (Broekhuis et al., 2020). This ontology defines eight main categories and 21 usability factors for eHealth usability (see Table 1). For each of the 21 usability factors we generated three items for our initial item pool. These 63 items were refined in several iteration rounds until we believed having obtained face validity. We started creating items by determining the themes per factor. For example, for the factor ‘fit between system and health goals’ we first formulated the following items: (1) I believe the system is helpful to [prevent/diagnose/treat/monitor] [health condition], (2), The system helped me manage my health effectively, and (3) The system would be useful for my health and well-being. Next, we started refining these items, by making the formulation consistent (starting all items with: I believe this system…), being consistent in the tense (item 2: ‘helped me’ changed to ‘helps me’) and changing wording based on discussions between researchers MB and LvV. For item 3, this meant changing ‘health and well-being’ to ‘health goals’. Last, we tried to make each items as short as possible while preserving the consistency. This meant using an active form such as ‘The system helps me to‥’. We verified the items with an independent researcher and made adjustments if necessary. Last, we decided to make the Accessibility category optional, by adding three questions, to check whether people had a visual, physical or cognitive impairment before answering questions about this topic. Table 2 shows all 66 items. The benchmarking instrument was developed in English and via the forward-backward translation method with bilinguals [16] translated into Dutch. Finally, we accompanied each item with a 5-point Likert scale answering option (totally agree–totally disagree).

**Table 2:** Item pool.

Category	Factor	ID	Items
Basic System Performance	Technical performance	BSP1	*The system is slow*
		BSP2	*The system interpreted my (health) data incorrectly*
		BSP3	*I experienced system errors*
	General system interaction	BSP4	*I get stuck when using the system*
		BSP5	*I understand how this system works*
		BSP6	*I find it difficult to use this system*
Task-Technology Fit	Fit between system and context of use	TTF1	*The system fits into my daily routine*
		TTF2	*The system is convenient to use at [home*, *hospital*, *care centre]*
		TTF3	*I cannot use the system pleasantly where I want to*
	Fit between system and user	TTF4	*The system is suitable for me*
		TTF5	*I don’t think this system is intended for me*
		TTF6	*I don’t see why I should use this system*
	Fit between system and health goals	TTF7	*The system is helpful to [inform about / prevent/diagnose/treat/monitor] [health condition]*
		TTF8	*The system helps me to manage my health effectively*
		TTF9	*The system is unsuitable for achieving my personal health goal(s)*
Accessibility		ACC1	*Do you have a visual impairment (such as colour blindness or poor vision)*?
			*If ’yes’*, *then items 17–19*. *If ’no’*, *skip these items*.
	Accommodativeness to perceptual impairments or limitations	ACC2	*I cannot use this system because of my visual or hearing impairment*
		ACC3	*It is easy to adjust settings to see or hear objects better in the system*
		ACC4	*The design of the system is suitable for people with a visual or hearing impairment*
		ACC5	*Do you have a physical impairment (such as problems with moving your fingers*, *wrist or arm)*?
			*If ’yes’*, *then items 21–23*. *If ’no’*, *skip these items*.
	Accommodativeness to physical impairments or limitations	ACC6	*I cannot use this system because of a physical health impairment*
		ACC7	*The system is considerate to users with a physical health impairment*
		ACC8	*My physical impairment makes it difficult for me to use this system*.
		ACC9	*Do you have a cognitive impairment (such as concentration or memory problems)*?
			*If ’yes’*, *then items 25–27*. *If ’no’*, *skip these items*.
	Accommodativeness to cognitive impairments or limitations	ACC10	*I cannot use this system because I have problems with concentration or my memory*
		ACC11	*The system requires too much mental effort from me to use*
		ACC12	*I feel that I cannot keep up with this system*
Interface Design	Design clarity	ID1	*I can see everything clearly in the system*
		ID2	*The objects in the system are too small for me to see*
		ID3	*I think the visual design of the system can be improved*
	Symbols, icons and buttons	ID4	*All buttons in the system have a clear function*
		ID5	*The signals*, *warnings and cues in the system are easy to interpret*
		ID6	*I don’t understand why some of the buttons or icons are there*
	Interface organization	ID7	*The information on each page is well organized*
		ID8	*The layout of each page is appealing*
		ID9	*The system has the same design everywhere*
	Readability of texts	ID10	*Text is easy to read*
		ID11	*Text size and lay-out make it hard to read*
		ID12	*The messages in the system are well-structured*
Navigation & Structure	Navigation	NS1	*I always know where I am when using the system*
		NS2	*I can easily go back and forth between different parts of the system*
		NS3	*I know where to find the information I need*
	Structure	NS4	*I found the system unnecessarily complex*
		NS5	*I understand the relationships among the different parts of the system*
		NS6	*I do not see why some parts of the system are there*
Information & Terminology	System information	IT1	*The system information is easy to understand*
		IT2	*I need more information about how to use the system*
		IT3	*The system clearly explains why standard procedures should be performed e*.*g*. *create account*, *log on*, *change settings*, *connect with other devices*
	Health-related information	IT4	*The system provides sufficient supporting health information*
		IT5	*The system uses medical terms that I am not familiar with*
		IT6	*The system offers clear explanations for difficult medical topics*
Guidance and Support	Error management	GS1	*If I make a mistake I can fix it easily*
		GS2	*The system error messages tell me how to fix problems clearly*
		GS3	*The system provides sufficient information to solve problems or mistakes*
	Procedural system information	GS4	*I am well guided through system procedures e*.*g*. *create account*, *log on*, *change settings*, *connect with other devices*
		GS5	*The system sufficiently explains how to perform system procedures e*.*g*. *create account*, *log on*, *change settings*, *connect with other devices*
		GS6	*I need more information about performing system procedures e*.*g*. *create account*, *log on*, *change settings*, *connect with other devices*
	Procedural health-related information	GS7	*The system provides sufficient information to support me in managing my health*
		GS8	*There is sufficient feedback to support me in managing my health*
		GS9	*The system instructs me properly on how to manage my health*
Satisfaction	Satisfaction with system	SAT1	*Overall*, *I am satisfied with this system*.
		SAT2	*I like this system*
		SAT3	*I would like to use this system more often*
	Satisfaction with system’s ability to achieve health goals	SAT4	*I like how this system contributes to my health*
		SAT5	*The system supports me in achieving my health goals*
		SAT6	*I believe this system is not suitable for [informing / preventing /diagnosing /treating /monitoring] [health condition]*

### Study procedure

An online study was set up to assess the internal reliability of the HUBBI and shorten the overall length of the questionnaire. This study consisted of three parts: (1) demographical questions about age, gender and education, (2) task scenarios related to thuisarts.nl (a Dutch website for informing the general public about common ailments and for instructing them when to call their General Practitioner or not) and (3) the HUBBI. Before participants filled out the HUBBI, they had to perform three tasks on the website. This was done to make sure they were familiar with the website before evaluating its usability. These were: (1) name the four factors that are mentioned as causes for Achilles tendinitis, (2), list the three medical specialists to which a general practitioner can refer you if you are suffering from sleep apnoea, and (3) find out how long it usually takes for brachial neuritis to heal. All participants agreed to participate by signing an online consent form before they took part in the online study.

### Participants and recruitment

People of 18 years or older, fluent in Dutch, were recruited to participate in this study. We recruited healthy participants via convenience sampling and a commercial panel agency situated in the Netherlands.

### Data analysis

Demographics and task performance were analysed with descriptive statistics. To test the internal reliability of the HUBBI, we assessed the quality of the measurement model via Partial Least Squares Structural Equation Modelling (PLS-SEM) in Smart-PLS [17]. We opted for PLS-SEM because (1) it allows one to test complex relationships between items, variables and constructs with a small sample size, (2) does not assume normal distribution of data, and (3) examines a theoretical framework by predicting the causal relationships of the constructs and variables in that framework [18]. The results helped us to understand the relationships between the constructs and the categories, in order to determine which items reflect each construct best. Ultimately, we used these insights to reduce the overall length of the HUBBI.

For conducting the PLS-SEM analyses, we followed the steps of Hair et al. [19]. For each category, we completed four phases: (1) creating the PLS-SEM measurement model, (2) checking for internal consistency, (3) assessing significance and relevance of formative indictors, and (4) assessing indicator strength. We explain each phase in full using the category ‘Basic System Performance’. Then, in the Results section, we list the outcome of the same procedure for the remaining categories.

#### Phase 1: Creating the PLS-SEM measurement model

**Fig 2:**
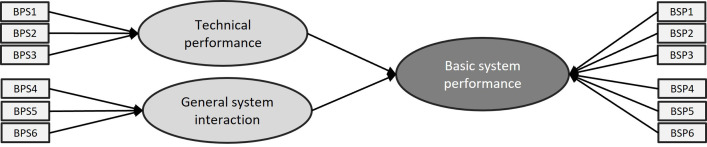
Formative Hierarchical Components Model (HCM) of the category ‘Basic System Performance’.

Each category can be represented by a formative Hierarchical Components Model (HCM, see Fig 2 here below). It consists of three components:

1. Higher-order component (HOC): this is the category ‘Basic System Performance’
2. Lower-order component (LOC): these are the constructs ‘Technical performance’ and ‘General system interaction’
3. Indicators: these are the items that belong to each construct (in this case, BSP1-BSP6).

Because we are dealing with a hierarchical model, almost all of the HOC variance will be explained by its LOCs. Any path coefficients (other than those by the LOCs) for relationships pointing at the HOC will be very small and insignificant. The solution for this is a two-stage HCM analysis. This type of analysis allows other latent variables that are not part of the HOC to explain some of its variance.

#### Phase 2: Checking for internal consistency

**Fig 3:**
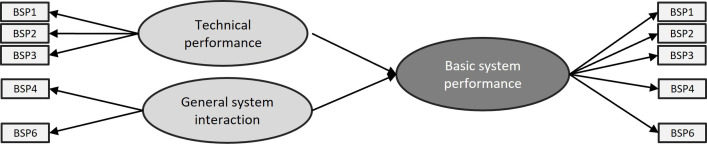
Reflective model of the category ‘Basic System Performance’ without item BSP5.

We switched the model from formative to reflective (see Fig 3). First, we checked the outer loadings per indicator. High outer loadings indicate that the associated indicators have much in common, which is captured by the construct. Every item should be above 0.7. If an indicator is below .7, then it is to be removed from the model. In this case, we had to eliminate item BSP5 from the model. Running the same test and again checking the outer loadings, it showed that all values are now ≥ .7, except for BSP1. This indicator has an outer loading of .669 in relation to the HOC, but a good value (.735) in relation to the LOC, therefore, we kept this indicator in for the time being.

Next, we checked the cross loadings. These values are an indicator of the discriminant validity and show the correlations of the indicators with the constructs. One would expect that items BSP1-BSP3 have a stronger correlation with the construct ‘Technical performance’ than with the construct ‘General system interaction’ and vice versa for items BSP4 and BSP6. If this is not the case, then the discriminant validity problem should be treated and established before continuing with the analysis. We immediately moved forward as there is no problem of discriminant validity. Next, we checked the following three measures for internal consistency: Cronbach’s alpha, composite reliability—a measure for internal consistency, similar to Cronbach’s alpha but not assuming equal indicator loadings, and the Average Variance Expected (AVE). Table 3 shows all values per measure. The first two measures should be ≥ .7 and the AVE should be ≥ .5. For constructs that had only one or two indicators (because of removal of indicators in the previous steps), we did not verify these measures. For example, in the category ‘Task-Technology Fit’, only one item was left for the construct ‘Fit between system and context’. Thus, the value of each measure for internal reliability was 1.

**Table 3:** Measures of internal consistency for the category ‘Basic System Performance’: Cronbach’s alpha, composite reliability and the Average Variance Expected.

Construct	Cronbach’s alpha	Composite reliability	Average Variance Expected
Basis System Performance	.832	.882	.601
Technical performance	.721	.843	.643
General system interaction	.704	.871	.771

#### Phase 3: Assessing significance and relevance of formative indictors

We switched the model back to formative (Fig 2, with item BSP5 removed from the model) and computed Outer Variance Inflation Factor (VIF) values, a measure of multicollinearity among the indicators in the formative measurement model. The VIF values should be ≤ 5 for each item, which they were. If this is not the case, then the collinearity issue should be treated before continuing with the analysis. Next, we ran a bootstrapping procedure with 5,000 bootstraps and checked the outer weights: the primary criterion to assess the relative importance of each indicator. The p-value for each indicator should be ≤ .05. If the item is not significant, we checked the formative indicator’s outer loading (no bootstrapping). If the outer loading is ≥ .5, then we kept the indicator in the model even if it is not significant. If the outer loading is < .5, the significance of the formative indicator’s outer loading needs to be checked and potentially be removed from the model. In this case, we kept BSP1 in the model since the outer loading is > .5.

#### Phase 4: Assessing indicator strength

**Fig 4:**
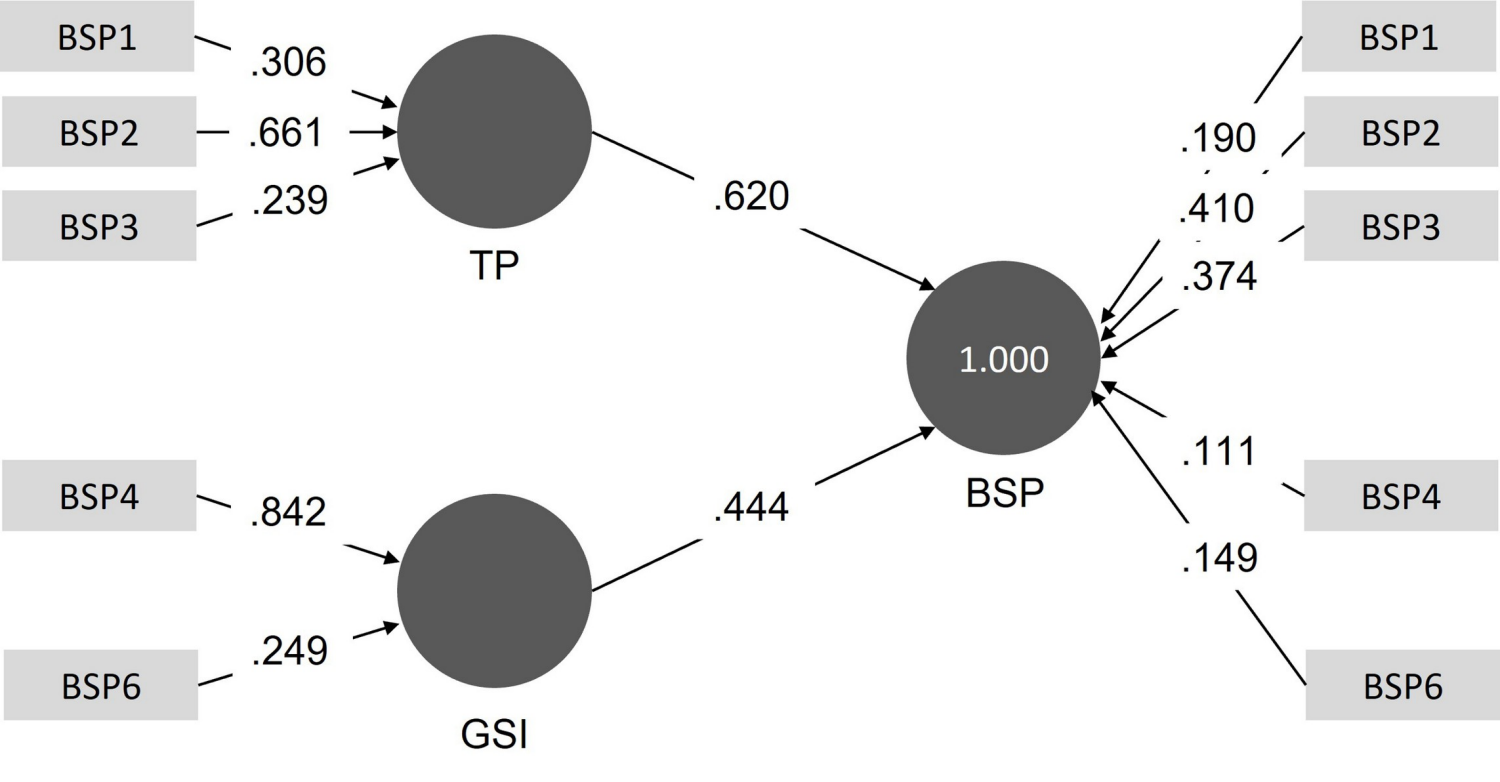
Path analysis of the category ’Basic System Performance’.

We ran the PLS Algorithm path analysis, based on the bootstrapping values (5,000) in the previous phase. The outcomes of this analysis are the path values per indicator. Fig 4 shows what this path model looks like. This model shows that for the construct ‘Technical performance’ (TP), indicator BSP3 had the highest value, which means that this indicator explains the most of the variance of the construct ‘Technical performance’. For the construct ‘General system interaction’ (GSI), this is indicator BSP4. Based on these results, we decided to only keep BSP3 (*I experienced system errors)* and BSP4 (*I get stuck when using the system*) in the final version of the HUBBI.

When reporting the results in the next section, we highlight those data analysis elements that led to the exclusion of an item. We report all path-values from item to the construct to the category, because these values determined which items were included in the final benchmarking instrument, namely the item with the highest value per construct provided that this value was significant.

## Results

### Demographics

A total of 148 people participated in this study. 109 participants were recruited via the commercial panel agency and 37 participants via convenience sampling. There were 68 (45.9%) male and 80 (54.1%) female participants with an average age of 50 years. 55.4% of these participants had completed a higher vocational education, 25.7% a vocational education, 16.2% a secondary education and 2.7% a primary education. Table 4 provides an overview of all demographical details.

**Table 4:** Demographics (gender, educational level) per age group.

Variabele		Age group				Total
		18–30	31–45	46–60	>60	
N		30	33	26	59	148
Gender	Male (N, %)	7 (4.7%)	16 (10.8%)	14 (9.5%)	31 (20.9%)	68
	Female (N, %)	23 (15.5%)	17 (11.5%)	12 (8.1%)	28 (18.9%)	80
Educational level	Primary education (N, %)	0 (0%)	0 (0%)	2 (1.4%)	2 (1.4%)	4
	Secondary education (N, %)	5 (3.4%)	1 (0.7%)	6 (4.1%)	12 (8.1%)	24
	Vocational education (N, %)	5 (3.4%)	8 (5.4%)	7 (4.7%)	18 (12.2%)	38
	Higher vocational education (N, %)	20 (13.5%)	24 (16.2%)	11 (7.4%)	27 (18.2%)	82

The majority of the participants (95, 64%) knew the website Thuisarts.nl and 83 participants (87%) of this group had previously used Thuisarts.nl. Most participants (55, 66%) used Thuisarts.nl once in the past three months, 20 participants (24%) used it once per month, five participants (6%) used it once per two weeks, two participants (2%) used it once per week, and only one participant (1%) used the website several times per week (1%).

### Task performance

#### Task 1—Achilles tendinitis

Of all 148 participants, 50 (33.8%) of them were able to successfully complete this task, listing all four correct answers. Another 50 (33.8%) participants listed three correct answers, 24 (16.2%) participants listed two correct answers, 15 (10.1%) participants listed one correct answer and 9 (6.1%) participants gave not one correct answer. This task was easy to complete according to 42 (28.4%) participants. 48 (32.4%) participants thought it was easy nor difficult and 58 (39.2%) participants thought it was difficult.

#### Task 2 –Sleep apnoea

90 (60.8%) participants were able to successfully complete this task, listing all three medical specialists. 12 (8.1%) participants mentioned two correct answers, 10 (6.8%) participants mentioned 1 correct answer and 29 (19.6%) participants gave no correct answer. There were also 7 (4.7%) participants that did not fill in anything. This task was easy to complete according to 101 (68.2%) participants. 32 (21.6%) participants thought it was easy nor difficult and 15 (10.1%) participants thought it was difficult.

#### Task 3 –Brachial neuritis

121 (81.8%) participants gave the correct answer and 27 (18.2%) participants gave a wrong answer on how long it usually takes for brachial neuritis to heal. This task was easy to complete according to 119 (80.4%) participants. 23 (15.5%) participants thought it was easy nor difficult and 6 (4.1%) participants thought it was difficult.

### Benchmark item selection

In this section, we highlight the main results per category. This resulted in an shortened version of the HUBBI, which can be found in S1 Appendix.

#### Basic System Performance

The category Basic System Performance consists of two constructs: technical performance and general system interaction. It included a total of six items, three items per construct. All items’ outer loadings were >0.7, except for BSP5, which we therefore excluded from further analyses. The resulting path values from the items to the latent variable were: BSP1 = .24, BSP2 = .31, BSP3 = .66, BSP4 = .84, and BSP6 = .25. Based on these results, we selected BSP3 and BSP4 for the final benchmarking tool.

#### Task-Technology Fit

The category Task-Technology Fit consisted of three constructs: fit between system and context of use, fit between system and user and fit between system and health goals. It included a total of nine items, three per construct. Outer loadings were >0.7, except for TTF1, TTF3 and TTF9. Therefore, we excluded these items from further analyses. We also removed one item (TTF5) after assessing the internal consistency. The outer weights were not significant for this item and it also had an outer loading of < .5. The resulting path values from the items to the latent variable were: TTF2 = 1, TTF4 = .73, TTF6 = .46, TTF7 = .74, and TTF8 = .43. Based on these results, we included TTF2, TTF4 and TTF7 in the final benchmarking tool.

#### Accessibility

The category Accessibility consisted of three constructs: accommodativeness to perceptual impairments or limitations, accommodativeness to physical impairments or limitations, and accommodativeness to cognitive impairments or limitations. It included a total of nine items, three items per construct. Each construct was optional, participants only answered the statements for each construct if they had a physical, perceptual or cognitive health impairment. Because of this, we unfortunately received insufficient data to conduct PLS-SEM analyses. The sample sizes per construct were too low for each construct: accommodativeness to perceptual impairments or limitations (*N =* 24), accommodativeness to physical impairments or limitations (*N =* 22), and accommodativeness to cognitive impairments or limitations perceptual limitations (*N =* 14).

#### Interface Design

The category Interface Design consisted of four constructs: design clarity, symbols, icons and buttons, interface organization and readability of texts. It included a total of twelve items, three items per construct. Outer loadings were all >0.7, except for ID6, ID9 and ID11. Therefore, we excluded these items from further analyses. The resulting path values of the items to their latent variable were: ID1 = .8, ID2 = -.15, ID3 = .55, ID4 = .55, ID5 = .59, ID7 = .51, ID8 = .59, ID10 = .44, and ID12 = .69. Based on these results, we included ID1, ID5, ID8, and ID12 in the final benchmarking tool.

#### Navigation & Structure

The category Navigation & Structure consisted of 2 constructs: navigation and structure. It included a total of six items, three items per construct. The outer loadings were all >0.7, so we kept all items for further analyses. The resulting path values from the items to the latent variable were: NS1 = .1, NS2 = .46, NS3 = .57, NS4 = .25, NS5 = .85, and NS6 = .03. Based on these results, we included NS3 and NS5 in the final benchmarking tool.

#### Information & Terminology

The category Information & Terminology consists of 2 factors: system information and health-related information. It included a total of six items, three items per construct. Outer loadings were >0.7, except for items IT2, IT3 and IT5. Therefore, we excluded these items from further analyses. The resulting path values from the items to the latent variable were: IT1 = 1, IT4 = .447, and IT6 = .68. Based on these results, we included IT1 and IT6 in the final benchmarking tool.

#### Guidance & Support

The category Guidance & Support consists of 3 constructs: error management, procedural system information and procedural health-related information. It included a total of nine items, three items per construct. The outer loadings were >0.7, except for item GS6. The resulting path values from the items to the latent variable were: GS1 = .1, GS2 = .72, GS3 = .29, GS4 = .45, GS5 = .59, GS7 = .15, GS8 = .54, and GS9 = .41. Based on these results, we included GS2, GS5 and GS8 in the final benchmarking tool.

#### Satisfaction

The category Satisfaction consists of 2 constructs: satisfaction with system and satisfaction with system’s ability to achieve health goals. It included a total of six items, three items per construct. The outer loadings were >0.7, except for item SAT6. The resulting path values from the items to the latent variable were: SAT1 = .48, SAT2 = .31, SAT3 = .39, SAT4 = .8, and SAT5 = .27. Based on these results, we included SAT1 and SAT4 in the final benchmarking tool.

#### Final HUBBI

The final, shortened, version of the HUBBI can be found in S1 Appendix. Here, only the 18 statements that were most significant in the path analysis are presented.

### Visualization of the HUBBI scores

**Fig 5:**
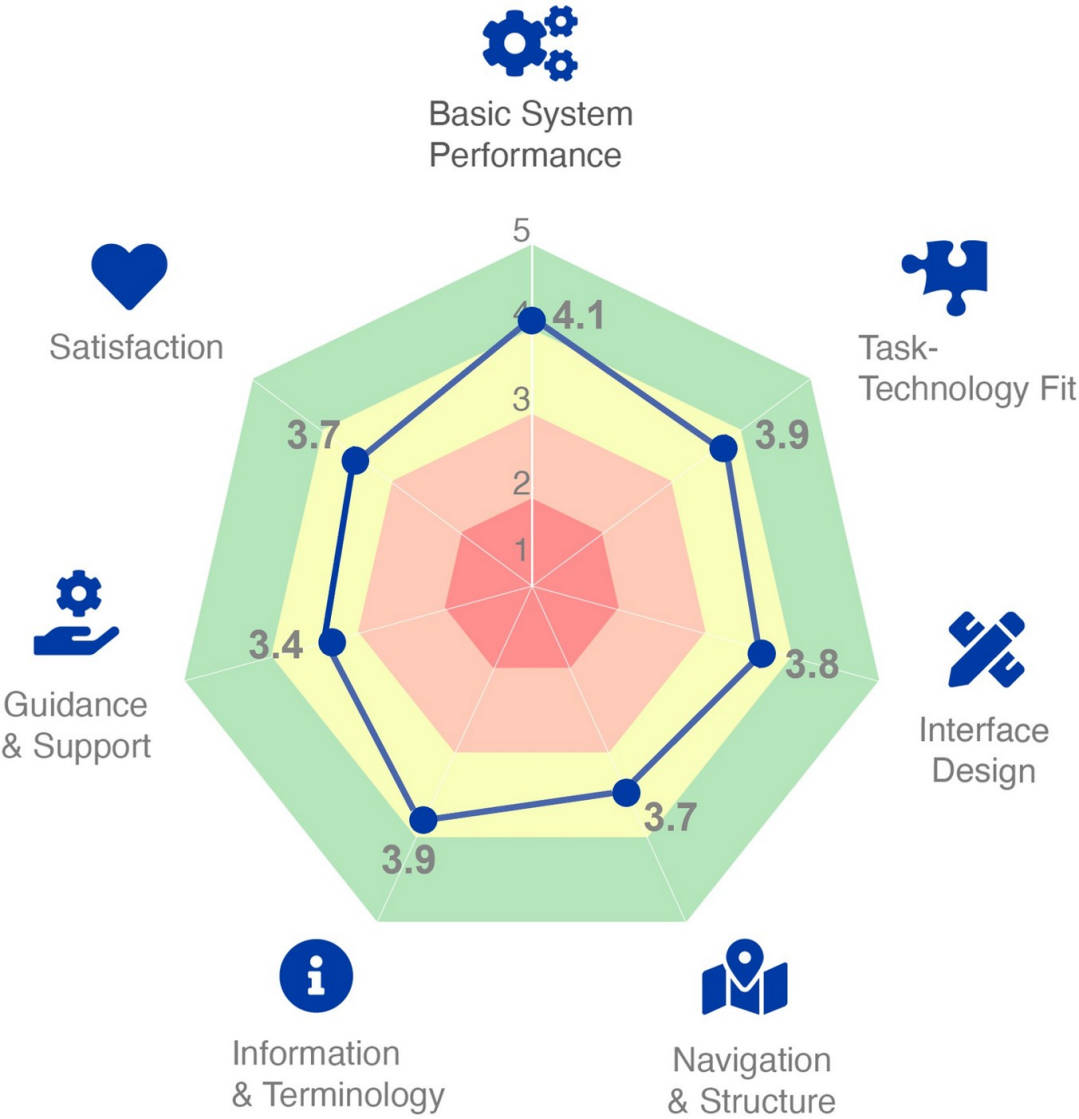
Visualization of the HUBBI using a radar chart.

**Fig 6:**
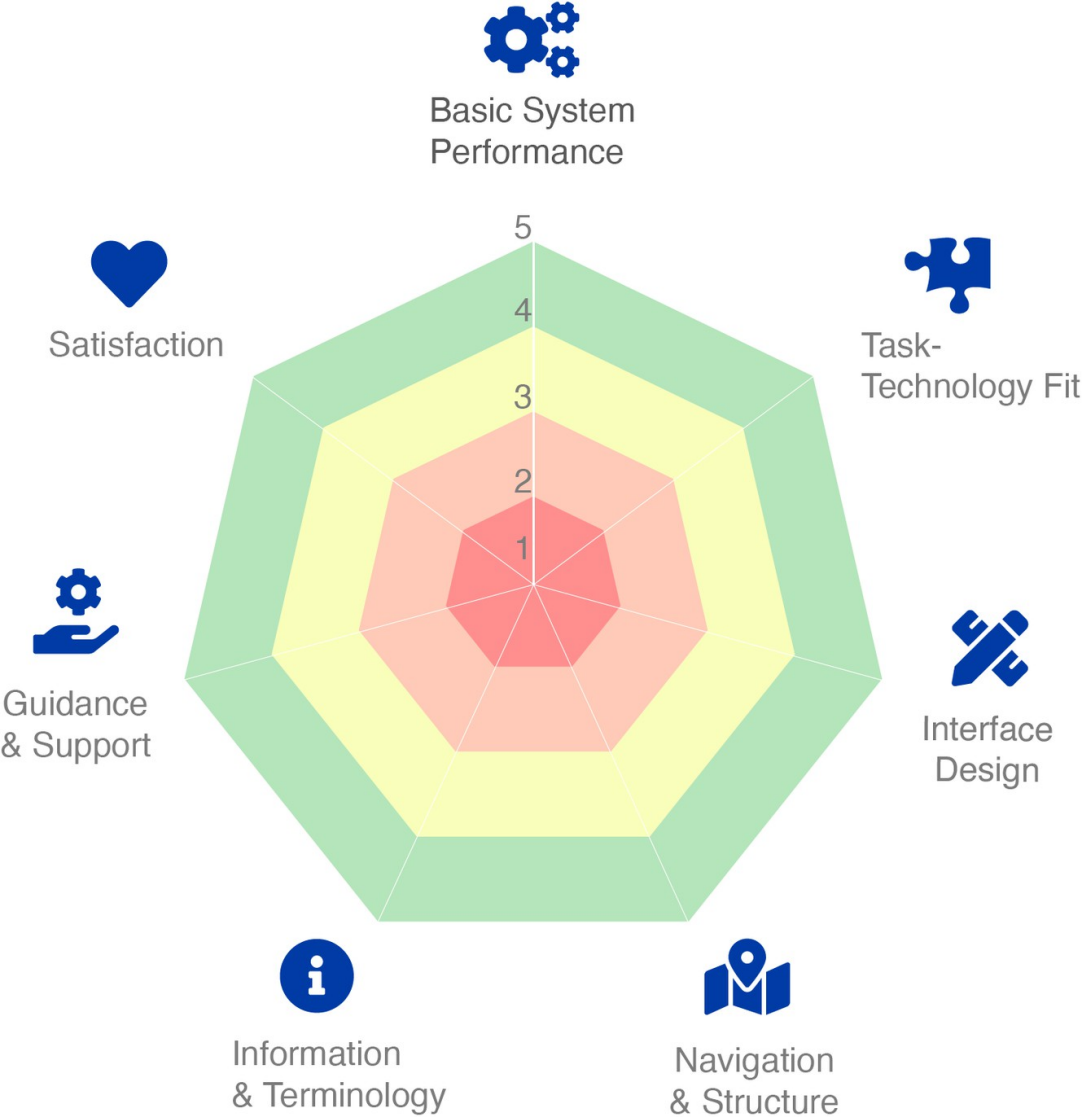


We approached the analyses of the HUBBI data on a category level. For easy interpretation of the HUBBI results, we recommend using a radar chart (see Fig 5). This figure shows the average results per category for the website Thuisarts.nl. This breakdown of the scores gives an immediate overview of what aspect of the system is lacking or thriving in usability. For example, in Fig 5 one can see that while the basic system performance of the system is quite good with a score of 4.1, on guidance and support, that has a score of 3.4, the system could improve its usability. S2 Appendix contains a blank version of the radar chart (Fig 6) for researchers and practitioners that want to use the HUBBI.

## Discussion

In this article, we have presented the development of the eHealth UsaBility Benchmarking Instrument (HUBBI), a usability benchmark that is specifically designed to deal with the intricacies of the eHealth domain. It consists of 18 items and a visualization method. The focus of this study was twofold: to verify the internal structure of the HUBBI and to reduce the number of items. The length of a survey is important to consider when developing a new survey. Research shows that when the length of the survey increases, the response rates and quality of the responses decrease [20, 21].

The sample size for the category ‘Accessibility’ was too low for any type of statistical analysis. Accessibility, while highly important to ensure inclusivity for all types of end-user groups, is a category that is not always (highly) relevant for an eHealth service. Granted, most eHealth services will need to serve end-users with this disability, but this group is too small, we realize, to warrant a specific sub-section in a general usability benchmark for the eHealth context. Therefore, we recommend to, when there are health impairments among the target audience to consider in the design of one’s eHealth system, to combine the HUBBI with a tool that is specifically designed to check the accessibility of such system. The golden standard here are the Web Content Accessibility Guidelines (WCAG) [22, 23].

Furthermore, we did not do an elaborate path analysis of all categories and constructs related to one overarching construct of ‘eHealth usability’. Instead, we conducted the analysis on category level. This decision was made because the HUBBI is based on an ontology that endeavours to capture the main aspects of eHealth usability that cause the most usability problems with eHealth systems, not those aspects that ‘make up’ eHealth usability. The HUBBI is therefore quite suitable as a benchmarking tool, but it is not a measurement scale for the overall usability concept.

### Comparison to other usability benchmarking instruments

What sets the HUBBI apart from other recently developed benchmarking instruments (see Table 5) is that (1) its categories, constructs and items are derived from common usability problems that are found in usability tests of modern-day eHealth systems, (2) it covers more categories of usability than other benchmarking instruments, and (3) it is an instrument that could be used for a wide variety of eHealth systems: it is not limited to eHealth systems that need to include specific functionalities.

**Table 5:** Characteristics of usability benchmarking instruments.

Usability benchmarking instrument	Year	Nr. of items	Answer options	Categories	Outcome
Questionnaire for User Interface Satisfaction (QUIS)	1988	27	9-point Likert scale	• Overall reaction to software • Screen • Terminology and system information • Learning • System capabilities	Interpretation of scoring for each individual item, that covers one facet of the system.
Post-Study System Usability Questionnaire (PSSUQ)	1992	16–19 (depending on version)	7-point Likert scale (strongly disagree-strongly agree + N/A)	• Usefulness • Information quality • Interface quality	Average of all items, or average per category.
System Usability Scale (SUS)	1996	10	5-point Likert scale (strongly disagree-strongly agree)	Undefined, items cover varies topics like ease of use, learnability, and intention to use	Single score of usability (0–100)
TeleHealth Usability Questionnaire (TUQ)	2016	17	7-point Likert scale (Disagree-Agree)	• Usefulness • Ease of use • Effectiveness • Reliability • Satisfaction	Averages per category
mHealth App Usability Questionnaire (MAUQ)	2018	16–20 (depending on version)	7-point Likert scale (completely disagree-completely agree)	• Ease of use • Interface and satisfaction • Usefulness	Averages per category
eHealth UsaBility Benchmarking Instrument (HUBBI)	2021	18	5-point Likert scale (strongly disagree-strongly agree)	• Basic system performance • Task-technology fit • Interface design • Navigation & structure • Information & terminology • Guidance & support • Satisfaction	Averages per category, plotted on a radar chart (see S2 Appendix)

Similar to other usability benchmarking questionnaires, the HUBBI uses a 5-point Likert scale and provides an average score per category. Additionally, the HUBBI has some overlap in terms of measurement items in other instruments, like the PSSUQ (*The system gave error messages that clearly told me how to fix problems*), MAUQ (*overall*, *I am satisfied with this system*) and Health-ITUES (*the information provided with [system] is clear)*. But while each of these questionnaires contain some elements of the HUBBI, they do not evaluate the full scope of *eHealth usability*, as covered in the ontology for eHealth usability [15]. In this ontology, 70% of the factors are general usability factors that are relevant to all digital technologies regardless of their specific domain. The other 30% are eHealth-specific factors that are essential for evaluating usability of eHealth applications. Likewise, in the work by Broekhuis et al. [15] an analysis of the usability issues identified within eight datasets was presented in which the division of general usability issues versus eHealth-specific usability issues (based on the ontology mentioned before) also displayed this 70%-30% split. The HUBBI reflects this 70%-30% division, as five out of the 18 items are eHealth-specific items while the remaining items are more generally formulated.

When looking at other usability questionnaires, it shows that the PSSUQ does not include any health-related items, the MAUQ does not include technical performance of the application nor the understandability of (medical) information in the app and the Health-ITUES does not include items on the fit between system and user, context or health goals nor items related to interface design. From the study of Broekhuis et al. [15] it became clear that these topics are of importance to evaluate the usability of eHealth services. The HUBBI, that is based on this ontology of eHealth usability, includes both those general and eHealth-specific usability factors that directly affect user interaction with an eHealth service.

Other differences between the HUBBI and other usability benchmarking questionnaires are that the SUS does not generate insights on which domains the technology can be improved. It provides only a single score without knowing what to improve if the score is low. Furthermore, the TUQ is designed specifically for eHealth services that include a videoconferencing module. This makes it a limited tool since not all eHealth services have such a module. Last, the QUIS is designed to measure primarily user satisfaction, which is just one domain of usability.

### Limitations

Of course, there are still issues to be resolved with the HUBBI. A limitation is that for this study on one eHealth system, an informational website has been used to assess the internal consistency of the HUBBI. That means that we currently lack insights to what extend the HUBBI is suitable for different types of eHealth systems. There are surely boundaries to the applicability of the HUBBI for eHealth systems to be expected. For example, serious games might not be completely suitable for the HUBBI as it does not include game-characteristics like game play, graphics, point-of-view and control [24]. It could be that for those cases, the HUBBI should be used in combination with game-specific evaluation instruments. More research on the HUBBI will provide better insights in the suitability of the HUBBI for eHealth systems in general. This research will consist of comparing the HUBBI to other (popular) usability parameters, like the System Usability Scale [2], task performance metrics and the number of usability issues derived from qualitative data collection methods, such as thinking aloud.

## Conclusions

This study presents a new alternative to outdated usability benchmarking instruments, specifically for the eHealth domain. We believe the HUBBI is unique in comparison to other benchmarking instruments, in the sense that it is based on an ontology of usability problems with modern-day eHealth systems. Further testing with the HUBBI is necessary to compare the HUBBI with other usability benchmarking instruments.

## Supporting information

S1 Appendix eHealth UsaBility Benchmarking Instrument (HUBBI). (DOCX)

S2 Appendix Visualization of the HUBBI (template). (DOCX)
