# Supplementary material for: Improving usability benchmarking for the eHealth domain: The development of the eHealth UsaBility Benchmarking instrument (HUBBI)
Source: PLoS One. 2022 Feb 17;17(2):e0262036. doi: 10.1371/journal.pone.0262036 (PMC8853524; doi:10.1371/journal.pone.0262036)
Supplement: S1 Appendix — (DOCX) [file pone.0262036.s001.docx]

# Appendix A: eHealth UsaBility Benchmarking Instrument (HUBBI)

| **Nr.** | **Statement** | **Answer option** |
| --- | --- | --- |
| 1 | I experienced system errors while using [the system] | Each statement should be answered on a 5-point Likert scale, ranging from:   1. totally disagree 2. disagree 3. Disagree nor agree 4. Agree 5. totally agree |
| 2 | I get stuck when using [the system] |  |
| 3 | [The system] is convenient to use at [home, hospital, care centre] |  |
| 4 | [The system] is suitable for me |  |
| 5 | [The system] is helpful to [inform about / prevent/diagnose/treat/monitor] [health condition] |  |
| 6 | I can see everything clearly in [the system] |  |
| 7 | The signals, warnings and cues in the system are easy to interpret |  |
| 8 | The layout of each page of [the system] is appealing |  |
| 9 | The messages in [the system] are well-structured |  |
| 10 | I know where in [the system] I can find the information I need |  |
| 11 | I understand the relationships among the different parts of the system |  |
| 12 | [The system] information is easy to understand |  |
| 13 | [The system] offers clear explanations for difficult medical topics |  |
| 14 | The error messages in [the system] tell me how to fix problems clearly |  |
| 15 | [ The system] sufficiently explains how to perform system procedures e.g. create account, log on, change settings, connect with other devices |  |
| 16 | [The system] provides sufficient feedback to support me in managing my health |  |
| 17 | Overall, I am satisfied with [the system] |  |
| 18 | I like how [the system] contributes to my health |  |

*Note: Because for readability and understandability, it is sometimes preferred to use the actual name of the system, like ‘Thuisarts.nl’ instead of ‘the system’. Because of this, we made slight alterations in statements 1 (BSP3), 2 (BSP4), 8 (ID8), 10 (NS3), 16 (GS8), 17 (SAT1) and 18 (SAT4), by changing ‘this system’ to ‘the system’ (2, 17, 18) , or adding ‘the system’ in the statement (1, 8, 10, 16). This improves the flexibility of the HUBBI for future research purposes by allowing researchers the possibility to add the name of the eHealth system in the statement of the HUBBI, if necessary. Especially for people with lower cognitive skills, children or specific vulnerabilities, this could be beneficial.*
