# Supplementary material for: Improving usability benchmarking for the eHealth domain: The development of the eHealth UsaBility Benchmarking instrument (HUBBI)
Source: PLoS One. 2022 Feb 17;17(2):e0262036. doi: 10.1371/journal.pone.0262036 (PMC8853524; doi:10.1371/journal.pone.0262036)
Supplement: S2 Appendix — (DOCX) [file pone.0262036.s002.docx]

# Appendix B: Visualization of the HUBBI (template)

**Fig 6. HUBBI scoring chart (template)**
